# The role of seed rain, seed bank, and clonal growth in plant colonization of ancient and restored grasslands

**DOI:** 10.1002/ece3.11611

**Published:** 2024-06-19

**Authors:** Rozália E. Kapás, Adam Kimberley, Sara A. O. Cousins

**Affiliations:** ^1^ Department of Physical Geography Stockholm University Stockholm Sweden; ^2^ UK Center for Ecology & Hydrology Lancaster UK

**Keywords:** ancient grassland, clonal growth, colonization potential, field experiment, grassland restoration, seed bank, seed dispersal, semi‐natural grasslands

## Abstract

Understanding the establishment of plant species is important to inform management of restored grasslands and to preserve biodiversity in ancient grasslands. In grassland communities, plant species can establish from seeds arriving via spatial dispersal, from seeds in the soil seed bank or through vegetative spread from nearby source individuals. However, this colonization potential and the likelihood of species establishment can vary in grasslands with different land‐use history. We investigated the relative importance of local species recruitment sources, such as dispersal in space and time and species presence in adjacent grasslands, in determining establishment of plant species in eight grasslands with different land‐use history (paired ancient grasslands continuously managed as pasture vs. restored grasslands on former forest). At each grassland, we established plots (0.25 m^2^) to monitor seedling emergence from seed dispersal, seed bank, and recorded clonal growth over two growing periods. We found that the likelihood of species establishment was highest from local seed rain, and that species present in the local species pool were more able to germinate and establish in both type of grasslands. Species from the seed bank and clonal growth contributed to a lesser extent to species establishment, but represented a greater proportion of the recolonization and regeneration of species in ancient grasslands. These results demonstrate that surrounding grasslands serve as a source for colonizing species and that dispersal from the adjacent grasslands is the key process in regeneration and colonization of plants. These results imply that the recovery of grasslands depends heavily upon to links to species source in grasslands, especially in restored grasslands. Therefore, management plans should incorporate rotational livestock grazing and larger networks of grassland in restoration efforts, which will enable to desirable species to establish and persist in grasslands.

## Graphical abstract

In our experiment we followed the assembly of the species over 2 years on different grassland types (ancient continuously managed vs. restored grasslands) with special focus on the origin of the species. We found that that the colonizing species are spatially dispersed and established from local species pools. Species from the seed bank mostly recruited in continuously managed grasslands rather than the restored grassland.

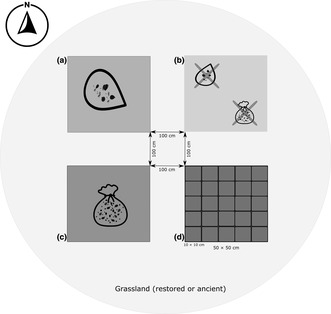

## 1 INTRODUCTION

Regeneration within plant communities, whereby adult plant individuals are replaced by younger individuals, has a great influence on plant population and community dynamics, and consequently in the maintenance of plant species richness (Grubb, 1977; Török et al., 2020). This is critical during plant assembly following restoration efforts, where the long‐term persistence of colonizing populations depends upon sufficient regeneration from the combination of reproduction from established individuals and continued incoming dispersal from neighboring populations (Evju et al., 2015; Hobbs et al., 2007; Kapás et al., 2023). Therefore, investigating mechanisms which facilitate plant species regeneration and colonization is fundamental in helping to design conservation and restoration measures (Kraft & Ackerly, 2014; Török et al., 2021). This is especially timely, with many current initiatives aiming to halt and reverse the ongoing decline in area of species‐rich grassland habitats, which is tightly linked to biodiversity loss (IPBES, 2019; UN, 2019).

Various mechanisms contribute to the success of the colonization and regeneration process, including the potential for species to disperse in space and time, and whether germination and establishment requirements of the species are met (Török et al., 2020). Hence, community assembly heavily depends on the abundance of and proximity to source populations at the landscape scale, the suitability of environmental conditions and the amount of available regeneration gaps for germination and establishment at the local scale (Kraft & Ackerly, 2014; Larson & Funk, 2016; Török et al., 2020; Zobel et al., 1998).

To be able to reach recipient communities through dispersal via seed rain, many plant species rely on vectors such as wind, water, or animals to disperse their propagules (Albert et al., 2015; Arruda et al., 2018). Dispersed seeds may immediately start to germinate after arrival where suitable conditions are present on site, ensuring that the available habitat remains occupied (Auffret et al., 2017). However, seeds are also able to integrate into the soil and build up a reservoir of seeds, which might support colonization and regeneration process in plant communities. These buried viable seeds might provide a future delayed establishment from the seed bank, when favorable conditions become present (Kiss et al., 2018; Plue et al., 2021). Besides dispersal from seed rain and the seed bank, plant species are also able to regenerate from vegetative shoots (i.e., clonal growth) via either bud bank stored in the soil (Ott et al., 2019) or lateral spread from nearby populations (Bullock et al., 1995; Johansson et al., 2011). These recruitment sources, for instance in the local pool next to restoration targets, might preserve a great diversity of plant species, which are able to support the regeneration process in both natural and degraded grassland habitats (Dzwonko & Loster, 1998; Milberg et al., 2019).

Direct and delayed seed dispersal and clonal growth therefore make different and independent contributions to species colonization and persistence in grassland habitats. However, the relative importance of these mechanisms may depend upon contextual factors, such as management or land‐use history and above‐ and belowground plant community composition at both local and landscape scales (Alexander et al., 2012; Vandvik & Goldberg, 2006). These factors may determine the ability of species to colonize following disturbances and the successful establishment of colonizers in both ancient and restored grassland habitats (Török et al., 2011, 2020). For instance, dispersal via seed rain might be vital for colonization and establishment of plant communities on heavily disturbed sites, where land‐use has altered the soil conditions or grazing has prevented the accumulation of seeds into soil (Saatkamp et al., 2014). Hence on these sites, seed bank driven establishment may contribute only a small extent to species presence (Bistea & Mahy, 2005; Klaus et al., 2018; Piqueray et al., 2015). Conversely, where disturbance or management is less intense, seeds are able to accumulate in the soil and therefore regeneration from the seed bank might act alongside dispersal of seeds from local propagule sources to better maintain the diversity and persistence of local populations (Jakobsson et al., 2006; Kalamees et al., 2012; Plue & Cousins, 2017). Consequently, long‐lived plant species on older and ancient sites may invest in producing vegetative clones rather than producing seeds to establish (Johansson et al., 2011; Ott et al., 2019).

Understanding the role of seed rain, seed bank or dispersal via vegetative mode and their influence on the presence of plant species in both restored and ancient grasslands is necessary to inform conservation of ancient grasslands and aid future planning of grassland restoration (Török, Helm, et al., 2018). These processes have been studied in greenhouse conditions, but few studies sought to directly investigate the underlying mechanisms of colonization or regeneration in ancient and restored grasslands (Bullock et al., 1995; Pakeman et al., 1998; Plue et al., 2021).

In addition, in situ experiments (i.e., our field germination study) may provide better insight about the potential for colonization and regeneration from seed rain or the seed bank contribution to the species assembly in grasslands under realistic settings (Jakobsson et al., 2006; Plue et al., 2017). Likewise, field germination study allows to follow the assembly of species under natural conditions (Jakobsson et al., 2006; Plue et al., 2017). Furthermore, most field studies quantify the recruitment sources of species on one or two grasslands (Török et al., 2011; Valkó et al., 2011), thus limiting their potential for understanding the role of local and landscape processes which may affect the recruitment and recovery success in grasslands. Hence, there is a need to study these processes across landscapes and range of grasslands (Arruda et al., 2018; Bakker et al., 1996).

In this study, we ask how much colonization and regeneration potential is provided through seed rain, seed bank and clonal growth, and investigate if there is a difference between restored and ancient grasslands and whether plant species presence in the adjacent species pool is important for species establishment. We compare germination and establishment of plant species in a field experiment over 2 years in recently restored grassland on former forest and livestock grazed and conservation‐managed grasslands across landscapes. Given the importance of proximity to nearby habitat for species diversity in grasslands, we expect that seed rain plays an important role in the presence of species in all grasslands. We expect seed bank and clonal growth to have a relatively higher contribution to regeneration and colonization in ancient compared to restored grasslands due to the higher population and possibly seed bank abundance for species in these sites. We also expect that this will be reflected in the community composition of emerged and colonized species in our experimental plots.

## 2 MATERIALS AND METHODS

### 2.1 Study sites

The study was performed in Södermanland County situated in southeastern Sweden (Table 1), the region is characterized mainly by forests and crop fields with fragments of ancient semi‐natural and restored grasslands (Cousins et al., 2002). Four sites were selected where both ancient and restored grasslands were present in close proximity to each other (Table 1). The restored grasslands had been overgrown for at least 60 years by trees and bushes which have been thinned or removed in the last 5 years (Table 1). The ancient grasslands have been managed as pastures or as meadows for many centuries, maybe even millennia (Cousins et al., 2002). All sites are within a 60‐km radius and have similar climatic and soil conditions and all are subjected to grazing. Detailed description of the sites can be found in Table 1.

**TABLE 1:** Overview of each grassland site used in the experiment with geographical location (WGS84), size of grassland, soil type, livestock type and grazing management, year of restoration and inventory of species pools per grassland type.

Site	Coordinates (Long, Lat)	Area (ha)	Soil type	Livestock	Grazing management	Year of restoration	Inventory of pool
*Tullgarn restored*	17°36.630′ E, 58°57.794′ N	4.34	Glacial silt	Cattle	Rotational grazing	2019	2022
*Tullgarn ancient*	17°36.874′ E, 58°57.863′ N	6.60	Clay	Cattle	Rotational grazing	‐	2022
*Övretorp restored*	16°55.446′ E, 59°3.370′ N	4.15	Rocky outcrop with shallow soil	Cattle	Rotational grazing	2018	2019
*Övretorp ancient*	16°55.310′ E, 59°3.437′ N	2.48	Clay	Cattle	Rotational grazing	‐	2020
*Nynäs restored*	17°24.263′ E, 58°47.696′ N	1.19	Rocky outcrop with shallow soil	Cattle and sheep	Rotational grazing	2017	2017
*Nynäs ancient*	17°24.478′ E, 58°47.702′ N	1.97	Rocky outcrop with shallow soil	Cattle and sheep	Rotational grazing	‐	2022
*Långmaren restored*	17°24.316′ E, 58°49.947′ N	5.22	Glacial silt	Cattle	Stationary grazing	2019	2022
*Långmaren ancient*	17°24.449′ E, 58°50.004′ N	6.01	Sandy morain	Cattle and sheep	Rotational grazing	‐	2022

*Note*: Three sites are restored within the European Union funded, Life Grace project.

### 2.2 Experimental set‐up and design

**FIGURE 1:**
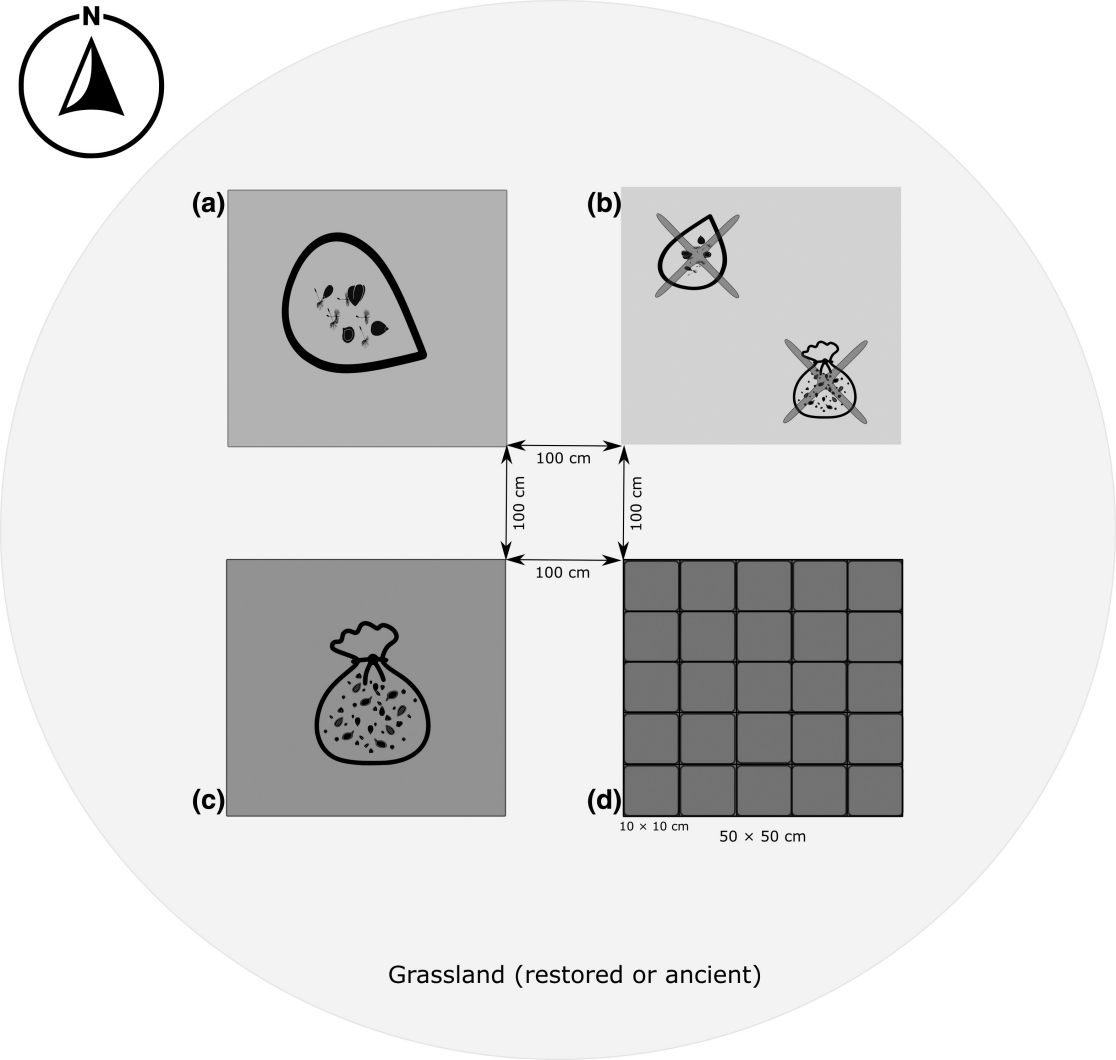
The experimental design for investigating plant species establishment in eight grasslands (four restored and four ancient) in Södermanland County, Sweden. We established four plots in each grassland (a) to allow seeds from the seed rain to establish on inverted soil surface. (b) We covered inverted plots to filter out potential remaining species from the seed bank or dispersed seeds in the soil. (c) We removed the top layer of the soil (e.g., disturbance gap) to monitor seedling emergence from seed bank. A control plot (d) in the standing vegetation was established for investigating the changes in plant community. During the seedling and clonal growth monitoring, we used a split‐up quadrat facing N‐S (on plot d) to follow the emergence and colonizing activity among species.

In each grassland, we established four 0.25 m^2^ (50 cm × 50 cm) plots 1 m apart from each other in summer of 2019 (Figure 1). Each plot was assigned to one of four experimental categories. In two of the four plots, we inverted the soil surface to a depth of ca.15 cm, exposing the lower soil layer (i.e., bare soil). This inversion of soil served as a competition free space for naturally occurring species to establish and aimed to eliminate the occurrence of seed banking species in the plots. One of these plots with the inverted soil acted as a potential establishment gap (seed trap) (Pakeman et al., 1998; Plue et al., 2017), and aimed to capture and allow to establish species arriving to the plot via seed rain from dispersal events covering all aspects of animal assisted (endo‐ or epizoochory) or unassisted (wind‐ or self‐dispersal) dispersal (Figure 1a). The other plot with inverted soil plot was covered by a metal mesh (size <0.05 mm) and termed as a covered seed trap to detect any potential remaining seed banking seeds in the inverted soil (Figure 1b). The mesh prevented seeds to establish from dispersal events thus, this plot served to filter out the potential remaining seed banking seeds in the soil and functioned as a negative control. In these plots, we found exclusively clonal growth, therefore we consider that the soil in the seed traps did not contain any seeds from the soil.

In the third plot, we created disturbance gaps on the surface by removing the top layer of vegetation (including litter, roots, and meristems) to expose buried seeds to light to induce germination of potential seed banking species and to allow plant species establishing from the seed bank at the start of the experiment (Figure 1c). After seed setting of the species (July–August), this plot also received species from the seed rain. The fourth plot was marked in the standing vegetation and not subjected to any experimental treatment, thus acting as a control (Figure 1d).

The set‐up resulted in a total of 32 plots in four pairs of restored and ancient grasslands. Prior to plot establishment in 2019 and during each census visit in the following years (2020, 2021) the vegetation was cut in 1‐m radius around each plot to prevent seed dispersal being dominated by species which happen to be in the immediate vicinity of selected plot locations. We established seed traps (inverted soil) in July 2019 to allow seeds to experience natural variation of temperature and light (i.e., cold stratification), while disturbance gaps to monitor species in the seed bank were initiated in April 2020.

### 2.3 Monitoring of species germination and clonal growth

We visited the experimental sites five times from April to October in 2020 and two times (July and November) in 2021. During each visit, emerged seedlings were identified using a quadrat facing in a north–south direction with 25 sub‐plots within each 0.25 m^2^ plot (Figure 1d). We identified and recorded each newly emerged seedling, in addition to the presence or absence of previously recorded seedlings and vegetative colonization (i.e., clonal growth) in each 10 × 10 cm sub‐plot. Seedlings were categorized as individuals with visible cotyledons (dicots) or seed hull (monocots) or seedlings that had already produced their first pair of leaves. Clonal growth was recorded as runners of plants from adjacent vegetation or re‐sprouting vegetation such as rhizomes or other belowground connections. In the seed bank plots, seedlings were counted and assumed to be seed bank driven seedlings in April, May, June, July, and August 2020. New seedlings in the plots were considered to be dispersal driven seedlings after August 2020 and included as species dispersed by seed rain in the analysis. This is because, in the studied grasslands the flowering and seed set peaks in end of July and beginning of August, therefore seedlings prior to this time must have been from the soil seed bank, while seedlings germinating after August likely to come from the seed rain than from the seed bank.

We were not able to identify eight species with 87 individuals that produced seedlings or clonal growth in the experiment due to insufficient identification characteristics. These individuals were removed from the main analysis, but included in the descriptive part. Furthermore, a small number of occurrences of species (52 individuals) were pooled together and treated as one genus in the analysis (see Tables S1 and S2). Nomenclature follows Mossberg and Stenberg (2010).

### 2.4 Species pool inventory in adjacent grasslands

We carried out species inventories to establish the local species pools for potential colonizers in surrounding the experimental plots. In each grassland, five plots (1 × 1 m, in total 40 plots) were distributed within a 100‐m radius from the experimental plots, one within 3 m from the experimental site and one at least 100 m away. The three remaining were placed randomly between these two plots. In each plot occurrence of all vascular plant species were recorded and additional species (not present in the plots) within the grassland were noted while walking (Table 1). In the modeling process, we used occurrences of species from the grassland which were also present in the experiment (i.e., species occurred in both the adjacent grassland and in experimental plots) to determine whether the species being recruited from the surrounding grassland to the experimental plots (local scale) or not.

### 2.5 Statistical analysis

In order to investigate the differences in the community composition of developing plant communities (seedling and clonal growth) among different treatments and grassland types, we performed a non‐metric multidimensional scaling ordination with Bray–Curtis distance (NMDS). We created a presence/absence species based matrix (species × treatment × grassland type) of the emerged seedlings (68 species × 20 plots) and the recorded clonal growth (113 species × 17 plots). To predict the probability of species occurrence as a seedling or clonal growth in different grassland types we used generalized linear mixed‐effects models with binomial family (estimated using ML and Nelder–Mead optimizer) on presence/absence of species occurred in the experiment. These models were ran on the list of species that germinated and/or exhibited clonal growth at least once during the experiment, because we aimed to generalize the observed variation in species presence response to seed recruitment sources across grassland types. We created two different models, one for recorded seedling and another one for clonal growth. In these two models, the presence or absence of species served as a response variable extracted from the species × treatment × grassland type matrix, while explanatory variables were treatment types (seed trap, seed bank, and control), grassland types (ancient or restored) and species presence or absence in the adjacent species pool (yes or no). The two models included species and individual grasslands as random effects. This means that the probability of occurrence for each species was modeled for each combination of treatment and grasslands, which allowed accounting for intrinsic differences in the potential of germination or clonal growth between species and grasslands, which is not related to treatments. The interaction between grassland type and treatment was included to investigate how origin of land‐use history influenced the likelihood presence of species in each recruitment sources (seed bank or seed rain), as this result has an implication for future restoration and conservation efforts in grasslands. However, we could not test this interaction among the clonal growth, due to the insufficient sample size in the model matrix.

All analyses were conducted in R (R Core Team, 2021) with package *vegan* (Oksanen et al., 2019), *lme4* (Bates et al., 2019) and model assumption and residual plots were visually checked by *sjPlot* (Lüdecke, 2022) and *DHARMa* (Hartig, 2022).

## 3 RESULTS

### 3.1 Seedling emergence and clonal growth presence throughout the sampling period

A total of 130 different plant species were identified among total 5122 emerged seedlings (2610) and recorded clonal growth (3381) in the two consecutive years (Table S1). We found 1814 seedlings from 71 species in restored sites and 796 seedlings from 59 different species in ancient sites. Among the emerged seedlings *Senecio viscosus* (558 individuals), *Stellaria graminea* (269) and *Campanula rotundifolia* (288) were the most abundant species on restored grasslands. On ancient grasslands, *Trifolium repens* (112), *Leucanthemum vulgare* (104), and *Viola* sp. (91) were the most frequently emerged species.

The three most abundant species that exhibited clonal growths were *Trifolium repens* (266), *Achillea millefolium* (185), and *Festuca ovina* (178) in all sites, but less species dispersed vegetatively in restored grasslands (45 species) compared to ancient (75). In the local species pool, that is, species occurring in each grassland, we found 111 species in total, 95 species in restored grasslands and 87 species in the ancient grasslands (Table S2). Restored sites were mostly inhabited by weedy and forest species while ancient grassland had a higher proportion of grassland species (i.e., species that tolerate regular disturbance from mowing or grazing animals). The three most frequent species in the species pool were *Trifolium pratense*, which were found 45% of the inventoried plots (19 of 40 plots) following by *Trifolium repens* (42%) and *Achillea millefolium* (35%).

**FIGURE 2:**
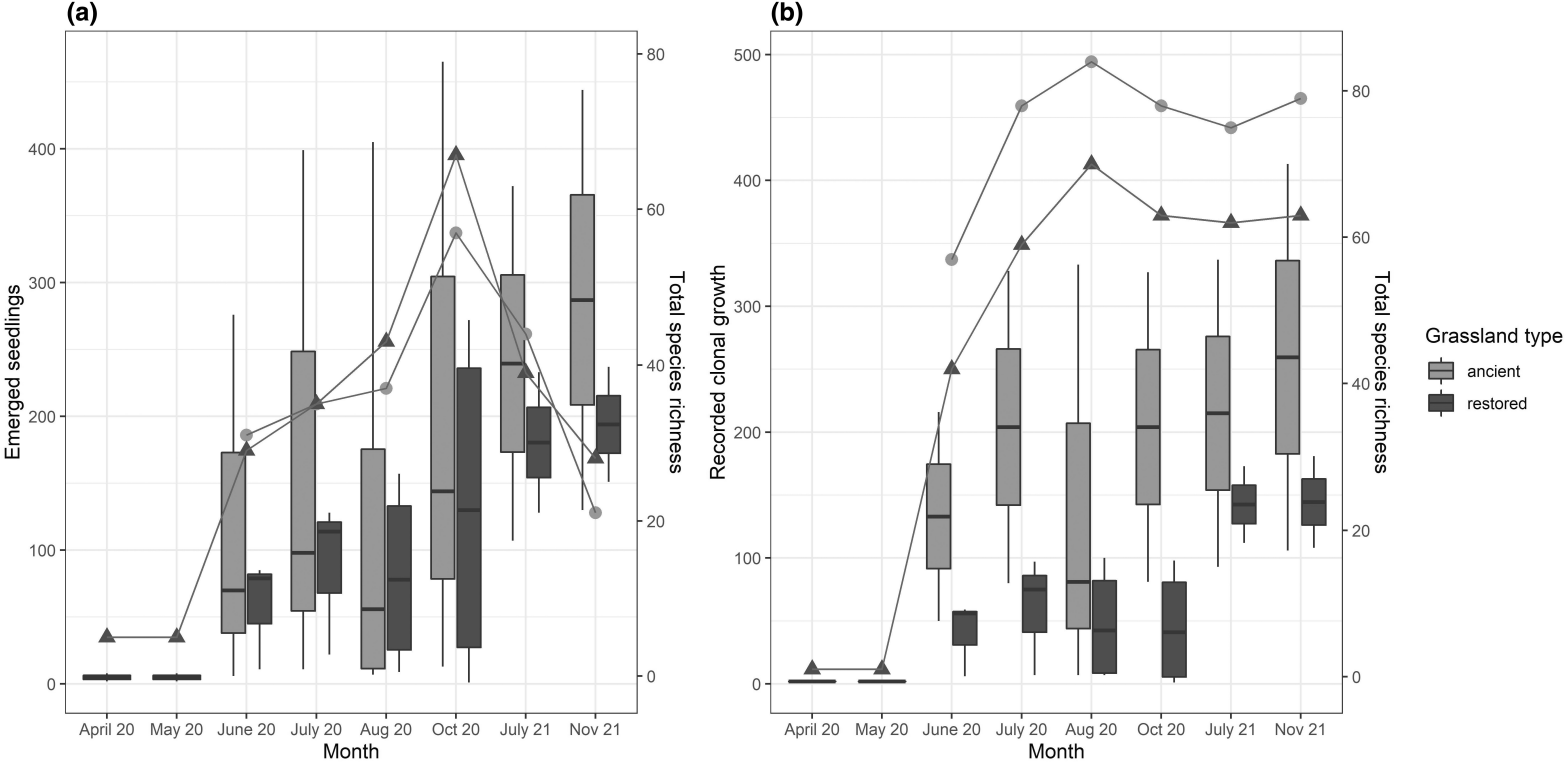
The relationship between the (a) number of emerged seedlings (b) recorded clonal growth and species richness in the experiment for two growing seasons in restored and ancient grasslands. Boxplots represent the upper and lower quartiles with median values for each month. Lines represent the changes in the species diversity, measured as the total species richness per month in ancient grasslands (dots) and restored grasslands (triangles).

There was a similar trend for seedling emergence on both grassland type; seedlings started to emerge in May with a small drop in August in the first year (2020) and peaking in late autumn in the second year (2021) (Figure 2a). The number of species among the emerged seedlings increased and most species occurred in the late season of the first year. However, fewer species were present in the second year. Similar trends could be seen among vegetative reproduction; clonal growth was increasing, for example, number of individuals that recruited from adjacent grassland or from seedling throughout the sampling period, but the species richness of the clonal growth reached plateau after the first year (Figure 2b).

### 3.2 Species composition among the emerged seedlings and recorded clonal growths

**FIGURE 3:**
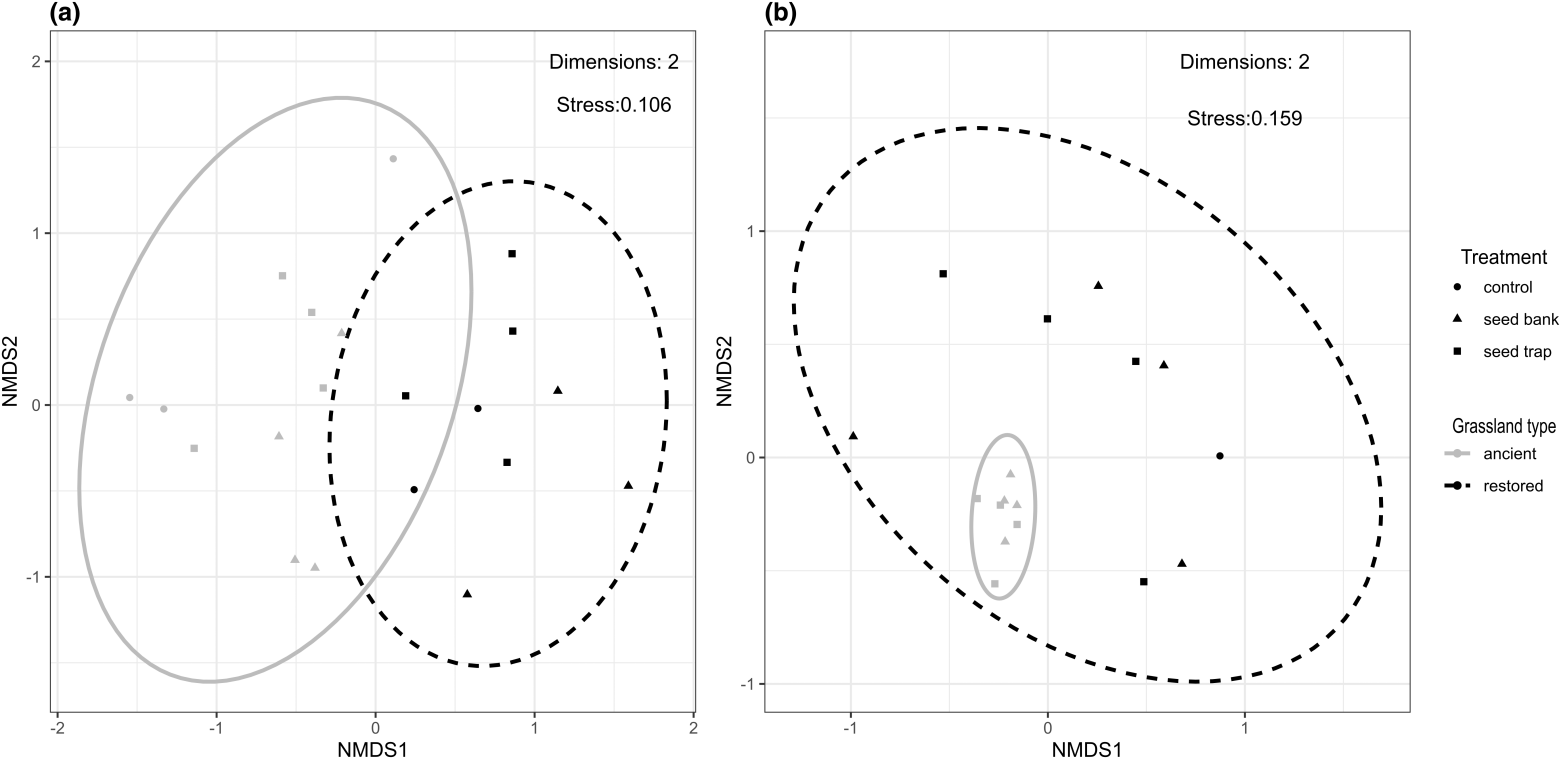
The similarity (NMDS ordinations) among (a) the emerged seedling communities and (b) recorded clonal growth on eight sites, including ancient and restored grassland grasslands in Södermanland County. Dots are the monitored experimental plots and lines represent the effect of different grassland types on the species composition based on the cluster of predictors.

The NMDS ordination showed that the species composition of the emerged seedlings was different between restored and ancient grasslands. However, communities from different treatment types were scattered in the ordination space (Figure 3a). This suggests that the restored and ancient grassland communities shared a small number of species, but there were no consistent trends with seed trap, seed bank, or control communities. Conversely, the recorded clonal growth communities from ancient grasslands were clustered within the restored grasslands communities (Figure 3b). This means that communities of clonal growth on ancient grasslands had very similar species composition to each other and it was subset of the restored grasslands.

### 3.3 Species presence on the different grasslands and recruitment sources

The probability of species presence as a seedling significantly differed among treatment (seed trap, seed bank, and control) and their interaction with grassland type (ancient and restored) (Table 2). More species established in the seed trap and seed bank in ancient grasslands (Figure 3). However, occurrence of species as seedling was higher in control plots of restored grasslands than in ancient ones (Figure 3a). Most seedlings that emerged in experiment were associated with species, which were also present in the local species pool. Hence, where species were present in grassland adjacent to the experiment, they occurred as seedlings in the experimental plots (Table 2). The interaction between plot type and grassland type had a negative effect on seedling presence in seed trap and seed bank of the restored grasslands, but not on the control plots, suggesting that the difference between the experimental treatments was greater in ancient grasslands (Table 2). This means that fewer species were able to colonize or regenerate in restored sites compared to ancient grasslands except for the control plots.

**TABLE 2:** Effects of grassland type (restored or ancient), plot type (seed bank, seed trap, and control), species presence in local species pool (yes or no) and interaction on the likelihood species presence as a seedling and clonal growth from the binomial generalized linear mixed models.

	Species presence as seedling	Species presence as clonal growth
Intercept	−3.905*** (−4.786, −3.023)	−1.891*** (−2.730, −1.051)
Grassland type: restored	0.737 (−0.434, 1.907)	−1.250*** (−1.849, −0.651)
Seed bank	0.989** (0.291, 1.687)	0.794* (0.067, 1.522)
Seed trap	2.008*** (1.376, 2.639)	0.256 (−0.478, 0.990)
Species present in pool: yes	0.809*** (0.511, 1.107)	1.002*** (0.741, 1.263)
Restored × Seed bank	−1.356** (−2.330, −0.382)	NA
Restored × Seed trap	−1.098** (−1.919, −0.278)	NA
Total model *R* ^2^	.32	.26
Fixed effects *R* ^2^	.13	.16

*Note*: Values for fixed effects are parameter estimates with lower and upper confidence intervals and *p‐values* indicated with asterisks. Significance values are the following: ***p ≤ .001, **.01 ≤ *p* < .001, *.05 ≤ *p* < .01.

The presence of clonal growth also significantly differed between grassland types and plot types and it was positively correlated with the species presence in local species pool (Table 2). Species were less likely colonize via vegetative dispersal in the seed trap (Figure 3b), and seed bank plots receiving most of the species. If species were available in surrounding grasslands, clonal growth were more pronounced in both ancient and restored grasslands (Table 2).

## 4 DISCUSSION

**FIGURE 4:**
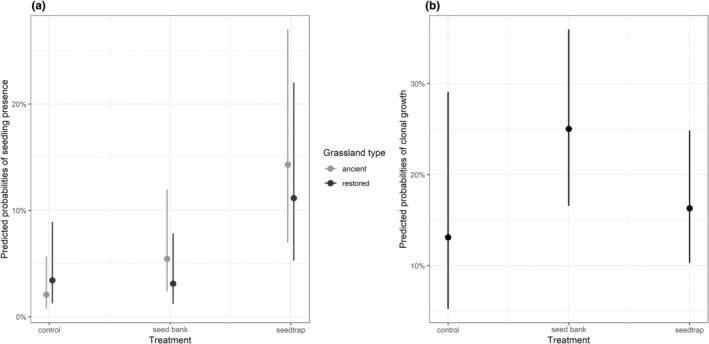
Predicted probabilities species as a (a) seedling and (b) presence of clonal growth in four pairs of ancient and restored grasslands from the binomial GLMM models. Dots represent median values for computed predicted values for experimental plot type and grassland from model predictors (marginal effects), while lines represent 95% confidence interval values. Significance values can be found in Table 2.

The different recruitment sources (i.e., seed rain, seed bank, and clonal growth) varied in their contribution to the species presence in ancient and restored grasslands (Table 2, Figure 4). Spatial dispersal of seeds contributed most to colonization and regeneration of species in both grassland types, and had a stronger positive effect when species were present in the adjacent species pool. Species dispersing via vegetative mode or species recruiting form seed bank were most likely to occur in ancient grasslands (Table 2, Figure 4b). This suggests that the environmental factors driving regeneration in restored grasslands may alter over time as plant communities develop. Furthermore, these results highlight that species in the seed bank can to some extent alleviate the reduced incoming seed dispersal in ancient grasslands, but spatial dispersal events from local propagule sources are essential for developing grassland communities in restored sites.

A sufficient pool of dormant seeds in the soil can support the regeneration of vegetation when seed dispersal is limited (Plue et al., 2021; Plue & Cousins, 2017). Our results show that in ancient grasslands, where time and land‐use has allowed the accumulation of seeds in the soil, seed bank has a greater contribution to the colonization and regeneration of plant communities (Kalamees & Zobel, 2002; Pakeman et al., 1998). Conversely, newly restored sites have been forested for a minimum of 60 years as in our study sites, this might have affected the germination potential of many seed banking and desirable grassland species, which became lost from the soil in the years prior to restoration (Bistea & Mahy, 2005; Bossuyt et al., 2006; Saatkamp et al., 2009).

We found that most of the species germinated from the seed rain (Figure 4) and in both grasslands with longer continuity of management (i.e., ancient) and restored grasslands received more species from spatial dispersal (Arruda et al., 2018; Conradi & Kollmann, 2016). This finding highlights that spatially dispersed seeds arriving to grasslands either via wind or mediated by animals tend to rule species establishment and early colonizers from seed rain are inevitable in colonization and regeneration of grassland communities regardless of land‐use history (Bakker et al., 1996; Bossuyt & Honnay, 2008; Piqueray et al., 2015). Further it highlights in restored grasslands, colonization is heavily dependent on the available species in the surroundings and the associated dispersal events (Conradi & Kollmann, 2016; Kapás et al., 2020).

Clonal growth was more common in ancient sites than in restored and strongest when the species were present in the adjacent grasslands (Figures 3b and 4b). Thus, dispersing via vegetative mode was a key mechanism of regeneration in ancient grasslands with long‐continuity of management (Johansson et al., 2011; Latzel et al., 2011). More clonal growth was observed in seed bank plots than in other plot types. One explanation could be that in these seed bank plots, the bud bank remained viable, despite initial disturbance during the experimental set‐up and providing an opportunity for many species to re‐sprout from this source (Latzel et al., 2011). Moreover, in these ancient grasslands a greater number of species/individuals in the surroundings were abundant and close enough to be able to colonize into disturbed gaps by clonal growth. As many grassland species have limited dispersal capability and they likely to invest more in clonal growth strategies (Cain et al., 2000; Lindborg, 2007). This was also evident in the studied ancient grasslands, where the vegetatively dispersed and established plant communities on the experimental plots composed of species that were very similar to each other (Figure 3b). This means that grasslands needs to be close to each other to allow species to establish via vegetative dispersing (Jakobsson et al., 2006). Moreover, it demonstrates that these species in ancient grasslands maintain their persistence with all three recruitment sources, which possibly complement each other at different levels challenging the conservation and restoration efforts.

In all experimental plots, there was a positive correlation between species which established and their presence in the adjacent species pool, which implies that species present in the local species pool were able to germinate and establish in the experimental plots (Table 2). Previous studies from the region showed a positive relationship between the number of emerged species in seed traps and seed banks and the abundance of species in the regional or local species pools, thus we assume this is the case in our experiment as well (Eriksson, 1997; Eriksson & Eriksson, 1997; Franzén, 2001; Jakobsson et al., 2006; Marteinsdóttir & Eriksson, 2014).

There was a clear difference in the plant identity and composition between restored and ancient grasslands of species establishing in experimental plots (Figure 3). In ancient grasslands, we found species typical for grassland communities (i.e., species that require regular disturbance to occur) such as *Trifolium repens*, *Leucanthemum vulgare*, or *Leontodon autumnalis* and these species colonized both seed bank plots and seed traps (Table S2). These species were present in all plot types, suggesting that their seeds were able to enter and later germinate from the soil, but also to disperse from sources occurring in the vicinity of the experiment. This shows that both processes contribute to survival of such species and highlights the persistence of species is complemented by both regeneration from buried seeds and colonization by spatial dispersal.

While ancient grasslands were abundant in species associated to grasslands communities (i.e., enduring regular disturbance), in restored sites we mostly noted early colonizers including annual and ruderal species such as *Cerastium spp*, *Senecio viscosus*, and *Stellaria graminea*. These species occurred mostly in the seed bank plots (Table S2). Species typically inhabiting heavily disturbed (e.g., after tree removal) sites often have long‐term persistent seeds or are effective wind dispersers (Dölle & Schmidt, 2009; van der Meijden et al., 1992), making them successful colonizers. This finding shows that seed banks only had species that are not typical for target grasslands communities after restoration took place (Godefroid et al., 2018; Török, Kelemen, et al., 2018).

Following restoration, degraded grasslands host a mixture of species associated with both forest and grassland communities (Jonason et al., 2014). This was also supported by our finding that seedlings establishing within restored grasslands shared some species with the ancient grasslands and with the previous land‐use (Figure 3a). However, forest species gradually disappear once grassland species have established (Dzwonko & Loster, 1998; Kapás et al., 2023). This could explain why we found more species in the species pool of restored grasslands (87 in ancient vs. 95 in restored grasslands) and there were higher probability for species to be present in the control plots of restored grasslands than in ancient ones (Figure 3a). On these restored sites it is common to have exposed and disturbed surfaces (i.e., environmental heterogeneity is high in plots with scarce vegetation) with many gaps allowing germination of seeds or integration to soil in a more efficient way, thus more species from the propagule source will be able to persist and survive (Grubb, 1977). This might be a short‐term phenomenon and possibly operates until the vegetation is established and become dense, hence less regeneration gaps will be available for arrival of seeds (Bullock et al., 1995; Kiss et al., 2021).

Difference in dispersal limitation may not be the only factor responsible for different colonization and regeneration patterns. Limited opportunities (e.g., lack of gaps in the vegetation) for establishment might have hindered successful colonization of some species (De Vitis et al., 2022; Eskelinen et al., 2022) or abiotic conditions (e.g., seasonal variation in precipitation and temperature) could have had strong impacts on recruits in the experiment (Moles & Westoby, 2004). First, seedling recruitment of certain species in the plots might have been negatively affected by the clonal growth density in year two (Figure 2b), which is resulted in greater number of emerged seedlings, but consequently lower species number in both grassland types (Figure 2a). This means only a few species could germinate and establish in the plots in the second year. Similar studies found that exclusion via competition by clonal growths and standing vegetation could be possible drivers behind seedling mortality of species with lower capability to survive with increasing competition for resources, hence outcompete target grassland species (Brown & Cahill Jr., 2020; Bullock et al., 1995; Marteinsdóttir & Eriksson, 2014). Another explanation could be that seedlings experienced a severe drought in the summer months of year which caused decline in the number of successful recruiters (Figure 2a). Increased clonal growth can result in suppression of desirable species, drought event can eventually halt the establishment and survival of desirable species (Eskelinen et al., 2022; Kapás et al., 2023; Tischew et al., 2014). These changes in conditions are likely to influence the persistence of species and the development of grassland communities to a greater extent on restored sites, where species have lower possibility from reproduce via clonal growth or regenerate from soil seed bank, hereby less chance to replenish failed establishment throughout the years (De Vitis et al., 2022; Eckhoff et al., 2023).

Although, our field germination study is limited in the number of plots, but it stretches across four landscapes, respective eight grasslands, thus aims at understanding the effects of different land‐use practices on source of plant species. In addition, we were able to follow the establishment of different species, hence the species germinated and were able to produce seeds, thus successfully colonized the plots. Despite the smaller sample size of our in situ experiment, results give an insight into developing grassland communities (i.e., assembly of species) under natural conditions (Jakobsson et al., 2006; Plue et al., 2017) and provide more realistic implications for variability in climate or disturbance (Hari et al., 2020; Kiss et al., 2018; Wilsey, 2021). With this knowledge restoration practices can ensure the successful establishment of species and long‐term persistence of grasslands. Our experiment also highlights that seed bank might not hold a great resource re‐colonization of grassland species in restored areas, but in ancient and managed grassland it can be a useful tool for restoration measures and accelerate the regeneration of grassland communities.

Our results show that at initial stage of species assembly restored grasslands are dependent on spatial dispersal until sufficient amount of species with self‐sustaining population are able to establish on them. It further highlights, when restoring grasslands and species‐rich species pools are not close, the restored grassland is likely to be colonized by fewer species in total, and not many typical grassland species, within a shorter time frame. In developing grasslands regeneration of species and the growth of local population fluctuate, which often results in temporary sink of species, however, as these grasslands age, they slowly turn into source population for such species. To boost the development of these grassland communities, restored grasslands should be linked with source of colonizing species via grazing livestock (i.e., functional connectivity), which can increase the dispersal of seeds and subsequently the establishment (Auffret et al., 2012; Kapás et al., 2020).

In conclusion, our results show that the dispersal of species from local species pool can greatly affect species establishment in grasslands and it is particularly important in restored grasslands, where soil seed bank or clonal growth have smaller contribution to colonization and regeneration pattern. In these sites, early colonizing species are more prone to extinction, due to lack of persistent and self‐sustaining population. Consequently, this might delay the recovery of grasslands, thus halt the species assembly in grasslands (Conradi & Kollmann, 2016; Öster et al., 2009). The results also highlight that managing existing or establishing new connection between source of species and target sites even across landscapes or via larger grassland network, is important when restoring grasslands (Bullock et al., 2002). Long‐term persisting grassland populations depend on a constant flow of seeds and to improve grasslands ability to recover from disturbances and reduce their vulnerability to extreme events, management should help to maximize target species availability from ancient grasslands and dispersal into restoration targets preferably via grazing animals (Brudvig et al., 2017; Eckhoff et al., 2023; Ladouceur et al., 2023; Schmid et al., 2017).

## AUTHOR CONTRIBUTIONS

**Rozália E. Kapás:** Conceptualization (lead); formal analysis (equal); investigation (lead); visualization (lead); writing – original draft (lead); writing – review and editing (lead). **Adam Kimberley:** Data curation (supporting); formal analysis (supporting); supervision (equal); writing – original draft (supporting); writing – review and editing (supporting). **Sara A. O. Cousins:** Conceptualization (equal); methodology (supporting); resources (lead); supervision (equal); writing – original draft (supporting); writing – review and editing (supporting).

## FUNDING INFORMATION

Oscar and Lili Lamm Memorial Foundation.

## CONFLICT OF INTEREST STATEMENT

The authors declare no conflicts of interest.

## Supporting information


Tables S1–S2

## Data Availability

Data used in the paper is available at Stockholm University's data repository (Figshare): https://doi.org/10.17045/sthlmuni.25650579.
