# Supplementary material for: The role of seed rain, seed bank, and clonal growth in plant colonization of ancient and restored grasslands
Source: Ecol Evol. 2024 Jun 19;14(6):e11611. doi: 10.1002/ece3.11611 (PMC11186710; doi:10.1002/ece3.11611)
Supplement: Supplementary file 1 — Tables S1–S2 [file ECE3-14-e11611-s001.docx]

**Supporting Information 1** Inventoried species and their frequency in the studied ancient and restored grasslands in southeastern Sweden.

| Species | Species frequency in the species pool (%) | Grassland type |
| --- | --- | --- |
| *Achillea millefolium* | 35 | ancient |
| *Achillea millefolium* | 5 | restored |
| *Achillea ptarmica* | 5 | ancient |
| *Agrostis capillaris* | 20 | ancient |
| *Agrostis capillaris* | 20 | restored |
| *Agrostis stolonifera* | 12,5 | ancient |
| *Agrostis stolonifera* | 5 | restored |
| *Ajuga pyramidalis* | 2,5 | ancient |
| *Ajuga pyramidalis* | 2,5 | restored |
| *Alchemilla sp* | 17,5 | ancient |
| *Alchemilla sp* | 5 | restored |
| *Alopecurus pratensis* | 12,5 | ancient |
| *Alopecurus pratensis* | 2,5 | restored |
| *Anemone nemorosa* | 2,5 | ancient |
| *Anemone nemorosa* | 10 | restored |
| *Anthoxanthum odoratum* | 22,5 | ancient |
| *Anthoxanthum odoratum* | 15 | restored |
| *Anthriscus sylvestris* | 5 | ancient |
| *Anthriscus sylvestris* | 2,5 | restored |
| *Anthyllis vulneraria* | 2,5 | restored |
| *Arrhenatherum elatius* | 2,5 | ancient |
| *Avenula pubescens* | 5 | ancient |
| *Avenula pubescens* | 12,5 | restored |
| *Bistorta vivipara* | 2,5 | ancient |
| *Briza media* | 17,5 | ancient |
| *Briza media* | 2,5 | restored |
| *Calamagrostis stricta* | 7,5 | restored |
| *Calluna vulgaris* | 2,5 | restored |
| *Campanula persicifolia* | 10 | ancient |
| *Campanula persicifolia* | 7,5 | restored |
| *Campanula rotundifolia* | 2,5 | ancient |
| *Campanula rotundifolia* | 7,5 | restored |
| *Carex canescens* | 15 | ancient |
| *Carex canescens* | 5 | restored |
| *Carex dioica* | 2,5 | restored |
| *Carex pilulifera* | 10 | ancient |
| *Carex sp* | 7,5 | ancient |
| *Carex sp* | 10 | restored |
| *Centaurea jacea* | 15 | ancient |
| *Cerastium fontanum* | 7,5 | ancient |
| *Cirsium arvense* | 5 | restored |
| *Cirsium palustre* | 7,5 | ancient |
| *Cirsium palustre* | 2,5 | restored |
| *Cirsium vulgare* | 5 | restored |
| *Cynosurus cristatus* | 2,5 | ancient |
| *Dactylis glomerata* | 15 | ancient |
| *Dactylis glomerata* | 5 | restored |
| *Dactylorhiza latifolia* | 2,5 | ancient |
| *Danthonia decumbens* | 12,5 | ancient |
| *Danthonia decumbens* | 5 | restored |
| *Daucus carota* | 5 | ancient |
| *Deschampsia cespitosa* | 30 | ancient |
| *Deschampsia cespitosa* | 10 | restored |
| *Deschampsia flexuosa* | 12,5 | ancient |
| *Deschampsia flexuosa* | 35 | restored |
| *Elytrigia repens* | 7,5 | ancient |
| *Elytrigia repens* | 5 | restored |
| *Festuca ovina* | 20 | ancient |
| *Festuca ovina* | 22,5 | restored |
| *Festuca pratensis* | 2,5 | restored |
| *Festuca rubra* | 12,5 | restored |
| *Filipendula ulmaria* | 10 | ancient |
| *Filipendula vulgaris* | 5 | restored |
| *Fragaria vesca* | 7,5 | ancient |
| *Fragaria vesca* | 20 | restored |
| *Galium boreale* | 22,5 | ancient |
| *Galium boreale* | 2,5 | restored |
| *Galium verum* | 17,5 | ancient |
| *Galium verum* | 5 | restored |
| *Gentianella campestris* | 2,5 | ancient |
| *Geranium pusillum* | 2,5 | ancient |
| *Geranium sylvaticum* | 2,5 | ancient |
| *Geranium sylvaticum* | 5 | restored |
| *Geum rivale* | 27,5 | ancient |
| *Geum rivale* | 2,5 | restored |
| *Gnaphalium sylvaticum* | 2,5 | restored |
| *Helianthemum nummularium* | 7,5 | ancient |
| *Helianthemum nummularium* | 5 | restored |
| *Helictotrichon pubescens* | 2,5 | ancient |
| *Hepatica nobilis* | 2,5 | ancient |
| *Hepatica nobilis* | 7,5 | restored |
| *Hieracium sp* | 2,5 | ancient |
| *Hieracium sp* | 7,5 | restored |
| *Hieracium umbellatum* | 2,5 | restored |
| *Hypericum perforatum* | 7,5 | ancient |
| *Hypericum perforatum* | 17,5 | restored |
| *Juncus conglomeratus* | 2,5 | restored |
| *Juncus effusus* | 2,5 | restored |
| *Juncus filiformis* | 2,5 | ancient |
| *Lactuca muralis* | 5 | restored |
| *Laserpitium latifolium* | 2,5 | restored |
| *Lathyrus linifolius* | 5 | ancient |
| *Lathyrus linifolius* | 22,5 | restored |
| *Lathyrus pratensis* | 15 | ancient |
| *Lathyrus pratensis* | 10 | restored |
| *Lathyrus sylvestris* | 2,5 | restored |
| *Leucanthemum vulgare* | 7,5 | ancient |
| *Lotus corniculatus* | 12,5 | ancient |
| *Lotus corniculatus* | 10 | restored |
| *Luzula campestris* | 2,5 | ancient |
| *Luzula campestris* | 2,5 | restored |
| *Luzula multiflora/campestris* | 2,5 | ancient |
| *Luzula pilosa* | 2,5 | ancient |
| *Luzula pilosa* | 22,5 | restored |
| *Luzula sp* | 2,5 | restored |
| *Melampyrum nemoralis* | 2,5 | restored |
| *Melampyrum pratense* | 2,5 | restored |
| *Melampyrum sylvaticum* | 2,5 | ancient |
| *Melica nutans* | 5 | restored |
| *Oxalis acetosella* | 2,5 | restored |
| *Phleum pratense* | 10 | ancient |
| *Pilosella officinarum* | 7,5 | ancient |
| *Pilosella officinarum* | 2,5 | restored |
| *Pilosella sp* | 2,5 | ancient |
| *Pimpinella saxifraga* | 12,5 | ancient |
| *Pimpinella saxifraga* | 7,5 | restored |
| *Plantago lanceolata* | 17,5 | ancient |
| *Plantago lanceolata* | 2,5 | restored |
| *Plantago major* | 2,5 | ancient |
| *Plantago major* | 2,5 | restored |
| *Poa pratensis* | 7,5 | ancient |
| *Polygala vulgaris* | 2,5 | ancient |
| *Polygala vulgaris* | 10 | restored |
| *Polygonatum odoratum* | 2,5 | restored |
| *Populus tremula* | 5 | restored |
| *Potentilla anserina* | 2,5 | ancient |
| *Potentilla erecta* | 12,5 | ancient |
| *Potentilla erecta* | 2,5 | restored |
| *Potentilla reptans* | 17,5 | ancient |
| *Potentilla reptans* | 10 | restored |
| *Primula veris* | 17,5 | ancient |
| *Primula veris* | 7,5 | restored |
| *Prunella vulgaris* | 27,5 | ancient |
| *Prunella vulgaris* | 2,5 | restored |
| *Prunus sp.* | 2,5 | ancient |
| *Pteridium aquilinum* | 7,5 | ancient |
| *Pteridium aquilinum* | 5 | restored |
| *Quercus rubus* | 2,5 | restored |
| *Ranunculus acris* | 30 | ancient |
| *Ranunculus acris* | 20 | restored |
| *Ranunculus repens* | 12,5 | ancient |
| *Ranunculus repens* | 7,5 | restored |
| *Rhinathus minor* | 7,5 | ancient |
| *Rosa sp.* | 2,5 | restored |
| *Rubus idaeus* | 12,5 | restored |
| *Rubus saxatilis* | 2,5 | ancient |
| *Rubus saxatilis* | 2,5 | restored |
| *Rumex acetosa* | 7,5 | ancient |
| *Rumex acetosa* | 5 | restored |
| *Rumex acetosella* | 2,5 | ancient |
| *Rumex acetosella* | 2,5 | restored |
| *Rumex sp.* | 5 | ancient |
| *Saxifraga granulata* | 2,5 | ancient |
| *Scrophularia nodosa* | 2,5 | restored |
| *Solidago sp.* | 2,5 | restored |
| *Stellaria graminea* | 22,5 | ancient |
| *Stellaria graminea* | 12,5 | restored |
| *Taraxacum sp.* | 2,5 | ancient |
| *Taraxacum sp.* | 5 | restored |
| *Trifolium campestre* | 2,5 | restored |
| *Trifolium medium* | 2,5 | restored |
| *Trifolium pratense* | 45 | ancient |
| *Trifolium pratense* | 10 | restored |
| *Trifolium repens* | 42,5 | ancient |
| *Trifolium repens* | 5 | restored |
| *Urtica dioica* | 2,5 | restored |
| *Vaccinium myrtillus* | 2,5 | ancient |
| *Vaccinium myrtillus* | 7,5 | restored |
| *Vaccinium vitis idea* | 2,5 | ancient |
| *Vaccinium vitis idea* | 7,5 | restored |
| *Veronica chamaedrys* | 15 | ancient |
| *Veronica chamaedrys* | 22,5 | restored |
| *Veronica officinalis* | 7,5 | ancient |
| *Veronica officinalis* | 20 | restored |
| *Vicia cracca* | 22,5 | ancient |
| *Vicia hirsutum* | 2,5 | restored |
| *Vincetoxicum hirundinaria* | 2,5 | restored |
| *Viola canina* | 7,5 | ancient |
| *Viola canina* | 7,5 | restored |
| *Viola riviniana* | 7,5 | ancient |
| *Viola riviniana* | 25 | restored |
| *Viola sp* | 2,5 | restored |

**Supporting Information 2** The number of emerged seedlings and clonal growth throughout the two years of experiment in the studied ancient and restored grasslands in southeastern Sweden.

| Species | Grassland type | | Number of seedling | | Number of clonal growth | |  |
| --- | --- | --- | --- | --- | --- | --- | --- |
| *Achillea millefolium* | | ancient | | 16 | | 185 | |
| *Achillea ptarmica* | | ancient | | 0 | | 4 | |
| *Agrostis capillaris* | | restored | | 1 | | 0 | |
| *Agrostis stolonifera* | | ancient | | 4 | | 94 | |
| *Agrostis stolonifera* | | restored | | 54 | | 64 | |
| *Ajuga pyramidalis* | | ancient | | 10 | | 0 | |
| *Ajuga pyramidalis* | | restored | | 10 | | 3 | |
| *Alchemilla sp* | | ancient | | 0 | | 18 | |
| *Anemone nemorosa* | | ancient | | 0 | | 13 | |
| *Anemone nemorosa* | | restored | | 0 | | 32 | |
| *Anthoxanthum odoratum* | | ancient | | 0 | | 35 | |
| *Anthoxanthum odoratum* | | restored | | 1 | | 3 | |
| *Anthriscus sylvestris* | | ancient | | 0 | | 1 | |
| *Arabidopsis thaliana* | | restored | | 2 | | 0 | |
| *Bistorta vivipara* | | ancient | | 0 | | 5 | |
| *Briza media* | | ancient | | 21 | | 57 | |
| *Calamagrostis arundinacea* | | restored | | 0 | | 27 | |
| *Calamagrostis sp* | | restored | | 0 | | 36 | |
| *Calamagrostis stricta* | | restored | | 0 | | 13 | |
| *Calluna vulgaris* | | restored | | 5 | | 1 | |
| *Campanula persicifolia* | | ancient | | 3 | | 61 | |
| *Campanula persicifolia* | | restored | | 7 | | 34 | |
| *Campanula rotundifolia* | | ancient | | 16 | | 15 | |
| *Campanula rotundifolia* | | restored | | 272 | | 5 | |
| *Campanula sp* | | ancient | | 0 | | 1 | |
| *Capsella bursapastoris* | | restored | | 3 | | 2 | |
| *Carex sp* | | ancient | | 1 | | 12 | |
| *Carex sp* | | restored | | 1 | | 3 | |
| *Centaurea jacea* | | ancient | | 4 | | 26 | |
| *Cerastium arvense* | | ancient | | 3 | | 4 | |
| *Cerastium arvense* | | restored | | 79 | | 19 | |
| *Cerastium fontanum* | | ancient | | 7 | | 0 | |
| *Cerastium fontanum* | | restored | | 80 | | 0 | |
| *Cerastium sp* | | ancient | | 4 | | 6 | |
| *Cerastium sp* | | restored | | 10 | | 34 | |
| *Cirsium arvense* | | restored | | 5 | | 3 | |
| *Cirsium palustre* | | ancient | | 2 | | 0 | |
| *Cirsium sp* | | ancient | | 0 | | 1 | |
| *Cirsium sp* | | restored | | 11 | | 1 | |
| *Cirsium vulgare* | | restored | | 0 | | 2 | |
| *Dactylis glomerata* | | ancient | | 0 | | 3 | |
| *Danthonia decumbens* | | ancient | | 3 | | 31 | |
| *Danthonia decumbens* | | restored | | 0 | | 6 | |
| *Dead unidentified* | | ancient | | 29 | | 0 | |
| *Dead unidentified* | | restored | | 10 | | 0 | |
| *Deschampsia cespitosa* | | ancient | | 0 | | 17 | |
| *Deschampsia cespitosa* | | restored | | 0 | | 30 | |
| *Deschampsia flexuosa* | | ancient | | 4 | | 41 | |
| *Deschampsia flexuosa* | | restored | | 1 | | 83 | |
| *Festuca ovina* | | ancient | | 21 | | 110 | |
| *Festuca ovina* | | restored | | 14 | | 68 | |
| *Festuca pratense* | | ancient | | 0 | | 4 | |
| *Festuca sp* | | ancient | | 0 | | 57 | |
| *Festuca sp* | | restored | | 0 | | 27 | |
| *Fragaria vesca* | | ancient | | 3 | | 55 | |
| *Fragaria vesca* | | restored | | 1 | | 19 | |
| *Galium boreale* | | ancient | | 3 | | 14 | |
| *Galium sp* | | ancient | | 0 | | 2 | |
| *Galium sp* | | restored | | 21 | | 0 | |
| *Galium verum* | | ancient | | 3 | | 57 | |
| *Galium verum* | | restored | | 6 | | 0 | |
| *Geranium pratense* | | ancient | | 2 | | 0 | |
| *Geranium sp* | | restored | | 0 | | 6 | |
| *Geum sp* | | ancient | | 5 | | 18 | |
| *Geum sp* | | restored | | 14 | | 1 | |
| *Helianthemum nummularium* | | ancient | | 5 | | 18 | |
| *Helianthemum nummularium* | | restored | | 1 | | 0 | |
| *Helictotrichon pubescens* | | ancient | | 0 | | 10 | |
| *Hieracium sp* | | restored | | 9 | | 0 | |
| *Hypericum maculatum* | | restored | | 3 | | 0 | |
| *Hypericum perforatum* | | ancient | | 2 | | 10 | |
| *Hypericum perforatum* | | restored | | 24 | | 15 | |
| *Lactuca muralis* | | restored | | 3 | | 0 | |
| *Lathyrus linifolius* | | ancient | | 0 | | 68 | |
| *Lathyrus linifolius* | | restored | | 0 | | 11 | |
| *Lathyrus pratensis* | | ancient | | 0 | | 46 | |
| *Lathyrus sp* | | ancient | | 0 | | 0 | |
| *Lathyrus sp* | | restored | | 0 | | 3 | |
| *Leontodon autumnalis* | | ancient | | 57 | | 24 | |
| *Leucanthemum vulgare* | | ancient | | 104 | | 48 | |
| *Lotus corniculatus* | | ancient | | 29 | | 10 | |
| *Lotus corniculatus* | | restored | | 7 | | 14 | |
| *Luzula campestris* | | ancient | | 0 | | 18 | |
| *Luzula pilosa* | | ancient | | 1 | | 1 | |
| *Luzula pilosa* | | restored | | 1 | | 3 | |
| *Luzula sp* | | ancient | | 0 | | 72 | |
| *Luzula sp* | | restored | | 0 | | 5 | |
| *Moehringia trinervia* | | restored | | 0 | | 1 | |
| *Oxalis acetosella* | | restored | | 2 | | 2 | |
| *Phleum pratense* | | ancient | | 0 | | 3 | |
| *Phleum pratense* | | restored | | 0 | | 2 | |
| *Picea abies* | | restored | | 1 | | 2 | |
| *Pilosella officinarum* | | ancient | | 0 | | 25 | |
| *Pilosella officinarum* | | restored | | 0 | | 3 | |
| *Pilosella sp* | | ancient | | 13 | | 1 | |
| *Pimpinella saxifraga* | | ancient | | 2 | | 111 | |
| *Pimpinella saxifraga* | | restored | | 1 | | 2 | |
| *Pinus sylvestris* | | restored | | 10 | | 0 | |
| *Plantago lanceolata* | | ancient | | 1 | | 14 | |
| *Plantago major* | | restored | | 1 | | 0 | |
| *Plantago media* | | ancient | | 0 | | 1 | |
| *Poa nemoralis* | | restored | | 0 | | 9 | |
| *Poa pratensis* | | restored | | 0 | | 11 | |
| *Poa sp* | | ancient | | 1 | | 1 | |
| *Poa sp* | | restored | | 2 | | 0 | |
| *Poacea sp* | | ancient | | 7 | | 1 | |
| *Poacea sp* | | restored | | 45 | | 1 | |
| *Polygala vulgaris* | | ancient | | 12 | | 8 | |
| *Polygala vulgaris* | | restored | | 0 | | 1 | |
| *Polygala vulgarisrium* | | ancient | | 0 | | 0 | |
| *Polygonatum odoratum* | | restored | | 0 | | 1 | |
| *Polygonum aviculare* | | restored | | 1 | | 3 | |
| *Populus sp* | | restored | | 0 | | 8 | |
| *Potentilla argentea* | | ancient | | 4 | | 9 | |
| *Potentilla argentea* | | restored | | 0 | | 1 | |
| *Potentilla erecta* | | ancient | | 8 | | 26 | |
| *Potentilla erecta* | | restored | | 4 | | 5 | |
| *Potentilla reptans* | | ancient | | 6 | | 13 | |
| *Potentilla reptans* | | restored | | 2 | | 4 | |
| *Potentilla sp* | | ancient | | 0 | | 5 | |
| *Potentilla sp* | | restored | | 1 | | 0 | |
| *Primula veris* | | ancient | | 13 | | 30 | |
| *Primula veris* | | restored | | 1 | | 0 | |
| *Prunella vulgaris* | | ancient | | 14 | | 9 | |
| *Prunella vulgaris* | | restored | | 1 | | 0 | |
| *Prunus sp* | | ancient | | 0 | | 3 | |
| *Prunus sp* | | restored | | 6 | | 5 | |
| *Prunus vulgaris* | | ancient | | 0 | | 5 | |
| *Pteridium aquilinum* | | restored | | 0 | | 5 | |
| *Pulmonaria sp* | | restored | | 3 | | 0 | |
| *Ranunculus acris* | | ancient | | 6 | | 27 | |
| *Ranunculus acris* | | restored | | 1 | | 37 | |
| *Ranunculus repens* | | ancient | | 7 | | 38 | |
| *Ranunculus repens* | | restored | | 2 | | 20 | |
| *Ranunculus sp* | | ancient | | 6 | | 4 | |
| *Rosa sp* | | restored | | 0 | | 22 | |
| *Rubus caesius* | | restored | | 0 | | 7 | |
| *Rubus idaeus* | | restored | | 31 | | 37 | |
| *Rubus saxatilis* | | restored | | 0 | | 4 | |
| *Rumex acetosa* | | ancient | | 0 | | 6 | |
| *Rumex sp* | | ancient | | 26 | | 12 | |
| *Rumex sp* | | restored | | 29 | | 0 | |
| *Saxifraga granulata* | | ancient | | 3 | | 17 | |
| *Saxifraga granulata* | | restored | | 0 | | 2 | |
| *Senecio viscosus* | | restored | | 558 | | 28 | |
| *Sorbus acuparia* | | restored | | 0 | | 1 | |
| *Stellaria graminea* | | ancient | | 12 | | 8 | |
| *Stellaria graminea* | | restored | | 257 | | 39 | |
| *Stellaria sp* | | ancient | | 0 | | 1 | |
| *Stellaria sp* | | restored | | 8 | | 3 | |
| *Taraxacum officinale* | | restored | | 1 | | 9 | |
| *Taraxacum sp.* | | ancient | | 4 | | 2 | |
| *Taraxacum sp.* | | restored | | 28 | | 9 | |
| *Trifolium arvense* | | ancient | | 0 | | 2 | |
| *Trifolium arvense* | | restored | | 0 | | 3 | |
| *Trifolium pratense* | | ancient | | 24 | | 98 | |
| *Trifolium pratense* | | restored | | 19 | | 4 | |
| *Trifolium repens* | | ancient | | 112 | | 245 | |
| *Trifolium repens* | | restored | | 41 | | 21 | |
| *Trifolium sp* | | ancient | | 11 | | 13 | |
| *Trifolium sp* | | restored | | 5 | | 4 | |
| *Urtica dioica* | | restored | | 26 | | 22 | |
| *Vaccinium myrtillus* | | restored | | 0 | | 6 | |
| *Vaccinium vitis idaea* | | restored | | 1 | | 48 | |
| *Veronica chamaedrys* | | ancient | | 4 | | 20 | |
| *Veronica chamaedrys* | | restored | | 3 | | 17 | |
| *Veronica officinalis* | ancient | | 3 | | 16 | |  |
| *Veronica officinalis* | restored | | 2 | | 35 | |  |
| *Veronica serpyllifolia* | ancient | | 0 | | 3 | |  |
| *Veronica serpyllifolia* | restored | | 0 | | 7 | |  |
| *Veronica sp* | ancient | | 0 | | 5 | |  |
| *Veronica sp* | restored | | 7 | | 2 | |  |
| *Vicia cracca* | ancient | | 1 | | 42 | |  |
| *Vicia cracca* | restored | | 0 | | 3 | |  |
| *Vicia sp* | ancient | | 3 | | 14 | |  |
| *Vicia sp* | restored | | 2 | | 15 | |  |
| *Vicia tetrasperma* | ancient | | 1 | | 1 | |  |
| *Vicia tetrasperma* | restored | | 1 | | 6 | |  |
| *Viola canina* | ancient | | 4 | | 32 | |  |
| *Viola riviniana* | ancient | | 3 | | 13 | |  |
| *Viola riviniana* | restored | | 2 | | 13 | |  |
| *Viola sp* | ancient | | 91 | | 134 | |  |
| *Viola sp* | restored | | 37 | | 24 | |  |
